# Antifouling Strategies for Sensors Used in Water Monitoring: Review and Future Perspectives

**DOI:** 10.3390/s21020389

**Published:** 2021-01-08

**Authors:** Adrián Delgado, Ciprian Briciu-Burghina, Fiona Regan

**Affiliations:** DCU Water Institute, School of Chemical Sciences, Dublin City University, Dublin 9, Ireland; adrian.delgado2@mail.dcu.ie (A.D.); ciprian.burghina2@mail.dcu.ie (C.B.-B.)

**Keywords:** biofouling, instrumentation, sensors, biocides, mechanical cleaning methods

## Abstract

Water monitoring sensors in industrial, municipal and environmental monitoring are advancing our understanding of science, aid developments in process automatization and control and support real-time decisions in emergency situations. Sensors are becoming smaller, smarter, increasingly specialized and diversified and cheaper. Advanced deployment platforms now exist to support various monitoring needs together with state-of-the-art power and communication capabilities. For a large percentage of submersed instrumentation, biofouling is the single biggest factor affecting the operation, maintenance and data quality. This increases the cost of ownership to the extent that it is prohibitive to maintain operational sensor networks and infrastructures. In this context, the paper provides a brief overview of biofouling, including the development and properties of biofilms. The state-of-the-art established and emerging antifouling strategies are reviewed and discussed. A summary of the currently implemented solutions in commercially available sensors is provided and current trends are discussed. Finally, the limitations of the currently used solutions are reviewed, and future research and development directions are highlighted.

## 1. Introduction

The adhesion and growth of microorganisms at the interface between any non-sterile medium and a solid surface is a widespread phenomenon in most environments on Earth. The development of disturbing or unwanted biofilms on surfaces is a major problem due to the accumulation of biomass that causes reduced efficiency, contamination, corrosion and failure of engineered components. This process is often undesirable in synthetic materials and surfaces from a technological, health or economic perspective. Therefore, the undesirable biological adhesion and growth on surfaces has been called biofouling [1,2]. Biofouling in the aquatic environment shortens the life-time of immersed structures, increases fuel consumption of ships and affects the functioning and data quality of water sensors [3]. The biofilm attached to vessels is responsible for the transport of invasive species from one ecosystem to another [4,5,6].

To control this biofouling problem, different antifouling solutions, such as coatings, have been used throughout history. Many of these coatings incorporate biocides, which base their effectiveness on generalised and non-selective toxicity to marine organisms. The impact of these biocides on the environment has led to the creation of legislation to regulate their use [5]. For example, the environmental impact caused by tributyltin (TBT), included in antifouling coatings, was first banned on 525 m vessels in most industrialised countries [6] and subsequently a worldwide ban by the International Maritime Organization (IMO) for all vessels in 2008. Due to the increasing environmental scrutiny of copper (Cu^2+^) and co-biocides, there is a renewed interest in the economic impacts of fouling on ships and a greater effort to develop effective non-toxic coatings [7].

The requirement for effective antifouling solutions is not limited to the shipping industry and extends to infrastructure for renewable energy, telecommunications and ocean and coastal observations. In the context of ocean monitoring, biofouling has long been considered a limiting factor and is recognised as one of the main obstacles to autonomous environmental monitoring in aquatic environments [8,9]. Much of the equipment currently used to monitor coastal and ocean waters relies on sensors incorporated into various platforms like buoys, subsea moorings and surface and subsurface vehicles [10]. All immersed components, including operational components (membranes, optical windows and electrodes), housings and mooring components are subject to biofouling and prone to irreversible damage [11]. For a large percentage of deployed instrumentation, biofouling is the single biggest factor affecting the operation, maintenance and data quality. This is particularly true for coastal and marine deployments. The Alliance for Coastal Technologies has estimated that up to 50% of operational budgets are attributed to biofouling, depending on location and season [12]. Such costs are associated with shorter deployment periods, loss of data due to sensor drift, frequent maintenance requirements and a shorter lifespan of the instrumentation. With recent advances in electronics, power management and battery life, and communication, biofouling is the key factor limiting the length of time a water monitoring instrument can stay deployed, particularly in long-term, continuous monitoring applications [12,13]. With a projected increase in operational architectures at the regional and global scales, to provide a backbone for active networking of autonomous platforms and environmental observations, the role of effective antifouling strategies for in situ sensors is paramount. Despite the importance of protection against biofouling for in situ instrumentation, progress towards an ideal operational strategy has been slow. Although many techniques have been developed and tested in the last decade very few of them have been implemented commercially. In many cases, the strategies used have been developed for the mature shipping industry and applied in their original or altered form to the instrumentation. The most notable developments and advancements have come from industry, with the development of specialised, tailored design solutions.

This paper provides a review of commercially available sensors and their biofouling control strategies and requirements. The state-of-the-art established and under development antifouling strategies are reviewed. Specific strategies applied to aquatic instrumentation and potential strategies from other research fields are examined. A summary of the currently implemented solutions in commercial sensors is provided and current trends are discussed.

## 2. Fouling Overview

Materials immersed in water experience a series of biological and chemical processes, resulting in the formation of complex layers with attached organisms. This biofouling can be divided into microfouling and macrofouling. Microfouling includes the initial events that result in the formation of a biofilm while macrofouling describes the settlement and development of macroscopic species [14,15].

### 2.1. Mechanism of Biofilm Formation/Progression

**Figure 1:**
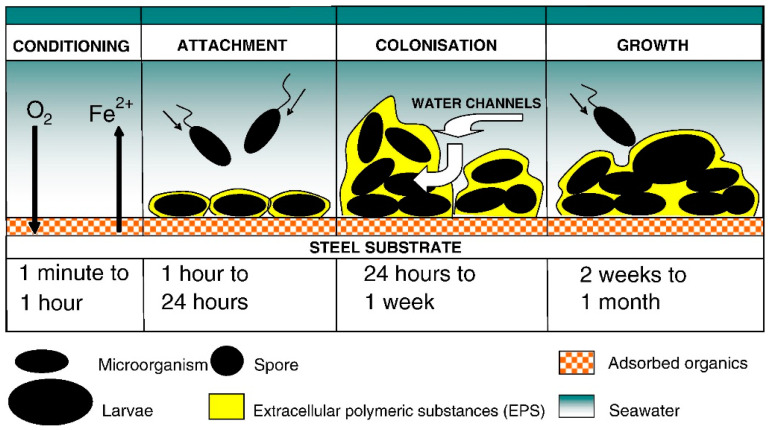
Illustration representing the stages of biofouling formation. Each of the stages shown can occur in the indicated order individually, in parallel or all at the same time [21].

It has been known since the early works of Zobell and contemporaries that aquatic life is concentrated at the interfaces [16,17]. The observations of Zobell and collaborators were noted for the initial insights gained on the mechanisms of microbial fixation on surfaces. Their research allowed for the understanding of biofilm formation and the processes that occur when a surface is immersed in an aqueous medium. It is now understood that the process of biofilm formation can be divided into several distinct stages (Figure 1). These stages depend on the length of time the surface has been exposed to the environment, the availability of nutrients and other environmental conditions such as temperature and pH.

The first chemical changes occur as the surface adsorbs the dissolved molecules and ions from the surrounding environment [18,19]. Specifically, the process can be divided into four stages. The first event is the adsorption of organic and inorganic macromolecules immediately after immersion, forming a film that covers the surface. The second event is the settlement of bacteria (microfouling) on the surface followed by a consolidation stage through the production of extracellular polymers, forming a microbial reversible film. Later, a more complex community (macrofouling) develops with the presence of multicellular species, microalgae, secretion of extracellular polymers (acid polysaccharides) and sediments adhering to the surface, producing an irreversible substrate as a polymeric bridge is formed between the cell and the substrate. Finally, the last stage corresponds to the fixation of larger marine invertebrates, such as barnacles, mussels and macroalgae. However, although numerous real-life experiments have been conducted with materials immersed in seawater, no specific fixation pattern can be differentiated for each of the biofouling stages [20]. These stages, unlike the ones mentioned above, do not occur in a completely staggered manner.

### 2.2. Properties of Biofilms

Bacterial biofilms represent an ancient prokaryotic survival strategy. This is because bacteria achieve significant advantages by providing biofilms for protection from environmental fluctuations in humidity, temperature and pH [22].

The ability to form biofilms does not seem to be restricted to any specific group of microorganisms and it is currently considered that under suitable environmental conditions the vast majority of bacteria, regardless of species, can exist by forming biofilms adhering to surfaces at a solid/liquid interface [22,23,24]. The biofilm matrix is made up of exopolysaccharides, which constitute its fundamental component, produced by the very microorganisms that make up the matrix. Macromolecules, such as proteins, nucleic acids and various products from the processes of bacterial lysis, are present in smaller quantities. The polysaccharides, nucleic acids and various other proteins are known as extracellular polymeric substances (EPS). Inorganic components, such as mineral salt crystals, corrosion particles and sediments, can also be found. They can have a neutral charge or polyanionic charge, depending on the type of exopolysaccharide, which would allow them to interact with different antimicrobials so that these can be trapped in the matrix without capacity to act on the bacteria [25].

EPS production is influenced by different environmental factors, such as water quality, temperature, depth and even season or geographical variation. These factors are relevant in the context of deployed instrumentation. Because local conditions vary so much, consideration should be given to the appropriate strategies for biofilm prevention on deployed systems. Many studies have shown strategies to prevent biofilm formation using mechanical, electrochemical or coating-based approaches. These are aimed at reducing the initial onset of the biofilm or active removal of a growing biofilm. These are addressed in detail later in the review.

## 3. Sensor Materials

Sensors can be immersed in the water at different depths. In order to operate in these environments, these systems must be built with materials resistant to chemical and biological corrosion and to wear and tear over time, so that the units can provide reliable information on a continuous basis. As the demand for sensors to be in contact with water increases, the selection of the correct materials for sensors depends on the proper selection of the appropriate alloys for the application and service environment. Table 1 outlines the types of materials that are used in sensing systems currently. The materials listed in the table are a summary of the types and sample applications of sensors. The entire sensor body is exposed to the water and therefore is liable to biofouling. Some manufacturers and researchers address only the measurement portion of the sensor when applying antifouling strategies and others address a whole sensor approach.

Whether corrosion is caused by varying depth levels, galvanic effects or biological attack, the first priority for good sensor performance over a long period of time is to match the right materials to the service application. Material selection is often affected by system reliability requirements, availability, cost and manufacturing capability.

In order to deter saltwater corrosion, manufacturers leverage superior materials. An example of this is the use of molybdenum in marine-grade stainless alloys, including 316 stainless steel alloy [28]. This type of material can be found in enclosures for portable power distribution systems designed exclusively for marine locations, industrial lighting products and more. Other types of marine-grade materials include the following: AH36, DH36 and EH36 (carbon steel); grade 5052 and 6061-T6 (aluminium); and C65500 (silicon bronze) [29,30,31].

Marine-rated devices for use in the marine environment on board ships or in submerged or exposed marine facilities and structures may also be treated with resilient coatings to ensure adequate protection from saltwater corrosion [11,32] Galvanization is a common method for achieving such features, which involves dipping the material or product in hot zinc [33]. Anodizing is a type of chemical treatment process using an electrolytic acid bath (highly applicable to aluminium). It is designed to strengthen the material, allowing it to withstand saltwater corrosion.

Advancements in material sciences have led to many manufacturers moving away from PVC and stainless-steel sensors due to the challenges in the operating environment. It has been seen that even materials such as steel can suffer corrosion processes produced by sulphate-reducing bacteria (SRB). These bacteria can produce hydrogen sulphide and acidic metabolites, such as acetic acid. It is suggested that the presence of hydrogen sulphide and acidic metabolites have a significant effect on the cathodic processes attacking these steel surfaces [34]. Therefore, new materials that can withstand salt water and biofouling, for example, polymers or titanium, are being used for sensors because they really increase the durability of the sensor. A trend towards the increased use of titanium is noticed among different sensor developers, such as Valeport (Totnes, Devon, UK) or YSI, a Xylem brand (Yellow Springs, OH, USA); others, like Turner Design (San Jose, CA, USA) in their C3 and C6-P models [35,36], use a delrin^®^ (Wilmington, DE, USA) housing, a highly crystalline thermoplastic (acetal resin) whose main advantages are its mechanical resistance and high hardness, ideal for subsea applications. The use of composite materials that combine the benefits provided by different materials can offer a high-performance solution. Such an example is carbon fibre-reinforced methylene polyoxide (POM), which combines the low friction, excellent wear properties and low water absorption of POM with the increase strength, stiffness and toughness provided by carbon fibres. An example of a custom-made housing, using a combination of reinforced glass fibre with POM, can be found in some specialized manufacturers, such as Develogic Subsea Systems (Hamburg, Germany) [37].

## 4. Antifouling Strategies for Sensors

**Figure 2:**
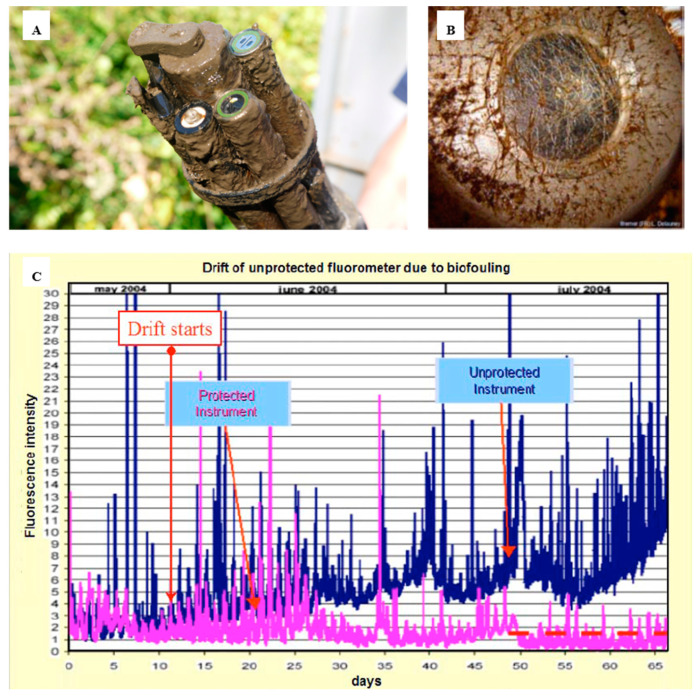
Overview of the biofouling on optical sensors and how this affects measurements over time. (**A**) EXO-sonde after it had been deployed (reproduced with permission of © 2020 YSI, a Xylem brand [38]); (**B**) transmissometer after 30–39 days in Throndheim harbour (Norway) during summer [11]; (**C**) drift of an unprotected fluorometer due to biofouling development on the optics [11] (© L. Delauney et al., 2010. This work is distributed under the Creative Commons Attribution 3.0 License).

Biofouling in the marine environment during the primary production period can proliferate, rapidly affecting the sensor head, therefore leading to measurement errors in a short period of time. Systems such cameras or optical sensors are also impacted by biofouling (Figure 2), generating visual artefacts, blurred images or noise, affecting the quality of the images and data. The problem is even more severe with optical sensors. Figure 2A illustrates the impact of even a short deployment of as little as one month on an optical sensor. The “slime” that forms has the potential to block the sensor signal and requires steps to remove it. In this case a mechanical wiper is attached. Figure 2B shows the optical element completely blocked. The data quality of the sensors that are impacted by a biofilm, where from an unprotected sensor, shows increasing data drift in Figure 2C. The properties of light are used to take the measurements; therefore, a slight deposit of biofilm on the optical elements can interfere with the measurements.

Sensors are made of many different materials and components and therefore antifouling strategies should be considered for all parts (Table 2). It is important to maintain a clean and easy-to-clean instrument during the deployment. More critical, in order to deploy a sensor over a long period of time, a metrological calibration must be performed before and after deployment. If the sensor housing is dirty, the instruments should be cleaned as soon as they are taken out of the water, especially if it is an optical sensor. These actions can change the state of the sensor’s sensitive area, making it difficult to compare the sensor’s metrological response before and after deployment.

### 4.1. Wiper Technologies

The simplest methods to remove biofouling from submerged structures such as boat hulls is the pressure cleaning of these structures with water, air or mechanical cleaning using brushes and wipers [60]. However, although these methods are simple and sometimes solve the problem of biofouling, they are not entirely feasible when applied to sensors with sensitive components.

**Figure 3:**
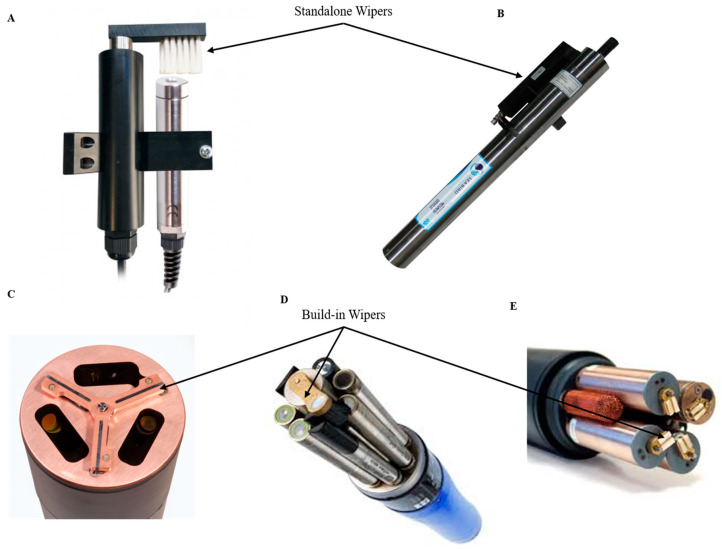
Standalone and build-in antifouling wipers overview. (**A**) Ponsel Dissolved Oxygen and Turbidity Sensors + antifouling wiper [62]; (**B**) SUNA V2 Nitrate sensor [63]; (**C**) ECO Triplet-w Sea-Bird Scientific sensor [64]; (**D**) YSI EXO 2 multi-parameter sonde [61]; (**E**) YSI 6 series multi-parameter sonde [65] (reproduced with permission of © 2020 YSI, a Xylem brand, Yellow Springs, OH, USA and Sea-Bird Scientific, Bellevue, WA, USA).

Wiper-based biofouling protection systems are purely mechanical methods and often they must be considered at the inception stage of sensor design. Some examples of mechanical wipers are shown in Figure 3. A mechanical antifouling system based on wipers must consider the material used in the wipers themselves, avoiding scratching the lens surface or other critical parts of the device. Its design should consider easy removal for replacement or repair as these often wear out. Some of these systems can be a sponge, offering a softer but less durable option than a brush as used in the YSI 6 series [52] (YSI, a Xylem brand, Yellow Springs, OH, USA). Many manufacturers of oceanographic instruments, such as the YSI EDS series (YSI, a Xylem brand, Yellow Springs, OH, USA), Hydrolab’s Self-Cleaning sensors (Loveland, CO, USA) or Wet Labs/Sea-Bird Bio (Bellevue, WA and Philomath, OR, USA), present built-in wipers working as a part of the mechanism of the sensor or independently as an accessory that is coupled to the measuring instrument (Figure 3). Due to the effectiveness of this strategy, many companies like Zebra-Tech (Nelson, New Zealand) specialize in the exclusive development of wiper technologies compatible with multiple probes, such as its Hydro-Wiper model [56]. The latter category functions as stand-alone wipers, which are often included as accessories and optional, coming at an extra cost. In terms of modularity, such wipers are designed to fit a wide range of sensors from the same manufacturer or they are specific to certain sensor versions. Although wipers are commonly used in commercial sensors, on-sensor power is required, which can limit the deployment duration. Damage due to abrasion can occur or macrofouling can obstruct movement. Today, a larger, fully integrated central wiper (YSI EXO-series [61], YSI, a Xylem brand, Yellow Springs, OH, USA) capable of cleaning all the sensor probes mounted on the sonde is the state of the art. The latter design coupled with a re-engineered conductivity probe allowed full reach of the mechanical wiper into the conductivity cell, providing fouling protection to previously vulnerable areas. In addition, integrated wipers require housing connection ports (plug-and-play wipers) or shaft ports that require waterproofing. Marine environments provide conditions for corrosion of waterproofed ports and connections. There is a need to ensure that the shaft, the motor and the electronics are properly sealed and protected against corrosion and water intrusion.

### 4.2. Biocide Generation Systems

The use of biocides, such as peracids, ammonium quaternary compounds and halogens, among others, can prevent the first stages of colonisation by the microfouling process. Sreenivasan and Chorny examined biocides and disinfectant foams on *Pseudomonas aeruginosa* biofilms, showing that aeration of a mixture of a foaming agent in conjunction with a common biocide was toxic to the bacteria making up the biofilm [66]. The use of foam disinfectants allows the reduction of the volume of biocides for disinfection. Another technique used in the cleaning of sensors is the use of chlorine and bromine solutions. These solutions are based on slow-dissolving chlorine (trichloroisocyanuric acid) and bromine have been used in closed optical systems to clean detection windows [67].

**Figure 4:**
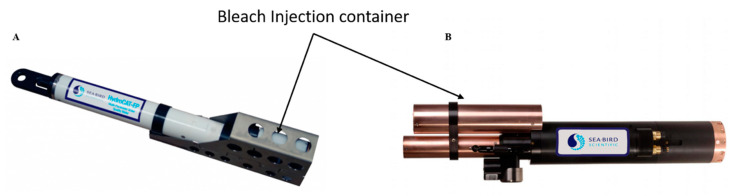
Bleach Injection container. (**A**) HydroCAT-EP, Sea-Bird Scientific [45]; (**B**) Water Quality Monitor (WQM) Wet-Labs and Sea-Bird Scientific sensor [50] (reproduced with permission of Sea-Bird Scientific, Bellevue, WA, USA).

Chlorination has been used for years in industrial applications to combat biofouling. Two modes of action are used, bleach injection and electrolytic chlorination. These methods are not widely used by manufacturers. Some (such as injection methods) can be found in freshwater monitoring stations and in autonomous monitoring systems like the WetLabs/Sea-Bird (Bellevue WA, and Philomath, OR, USA) WQM instrument (Figure 4). This figure shows the orientation of the bleach injection system on the sensor body. The reservoir contains approximately 125 mL of bleach that is pumped to the protected area as required.

This system is based on the injection of bleach into the conductivity cell, inhibiting the growth of microorganisms and prolonging the deployment for long periods of time. Because electrolysis chlorination strategies have very high energy requirements, very few commercial instruments are equipped with this type of strategy.

Davis and collaborators developed a method based on the use of solid bromine tablets placed inside a perforated container to reduce the effects of biofouling in optical systems [67]. This solution prevented the growth of biofouling, but it was difficult to maintain consistent concentrations. The effectiveness and usefulness of the chemical supply method for combating biofouling is unpredictable. Rajagopal et al. studied the response of the fouling hydroid *Cordylophora caspia* to chlorination [68]. They observed a complete degeneration in the growth rate of fouler organisms after 3 days with concentrations of 1 mg/mL of residual chlorine.

Protection based on TBT (tributyl-tin) leaching and paints for biofouling protection was extremely efficient. TBT was banned by the International Maritime Organization (IMO) in 2008. These compounds are considered to be highly toxic to the environment [69,70]. However, despite the ban, the American company Sea-Bird Scientific (Bellevue, WA, USA) continues to get approved by the Environmental Protection Agency (EPA) for this biocide [42,45,50]. Their strategy employs TBT rings in a pumping system coupled with a conductivity sensor. This way, when the conductivity sensor takes a measurement, the pumping system is switched on and its concentration is diluted. The pump flushes the sampled water and quickly moves a new sample into the flow path so that conductivity and oxygen measurements are more accurate. Water does not flow freely through the flow path so it stays saturated with the antifouling chemicals [45].

Following the ban of TBT-based products, alternatives containing copper (Cu)-based compounds were developed. Copper-based compounds that are less toxic than TBT, cobiocides, also called boosters, were used to enhance the antifouling performance of copper-based coatings [6]. Copper-based antifouling paint can be classified into two groups. The first group are slow-release films, releasing cuprous oxide into the surrounding environment by leaching. The second type are ablative antifouling paints that have a continuously toxic surface. The released bivalent Cu^2+^ interferes with the enzymes on cell membranes, avoiding cellular division [71,72]. However, some studies have shown that copper is not completely efficient as a biocide on its own, as some of the common marine algae have tolerance to this compound [73]. There is evidence of the diffusion of these compounds in many countries (Europe, North America and Japan) with significant concentrations of copper in marinas and harbours [7]. This has been shown to cause the generation of biocide resistance by bacteria, especially in estuarine environments where most ships and aquaculture structures are moored [74,75]. Because of this, this element must be used in combination with biocide reinforcements such as Irgarol^®^ 1051 (BASF, Ludwigshafen, Germany), Diuron^®^ (Bayer, Leverkusen, Germany), Zinc pyrithione, Sea-nine^®^ 211 (Dow Chemical Company, Midland, MI, USA), Dichlofluanid, Ziram, Thiram, Chlorothalonil, Kathon 5287 or Maneb/Zineb to be effective [76,77,78,79].

**Figure 5:**
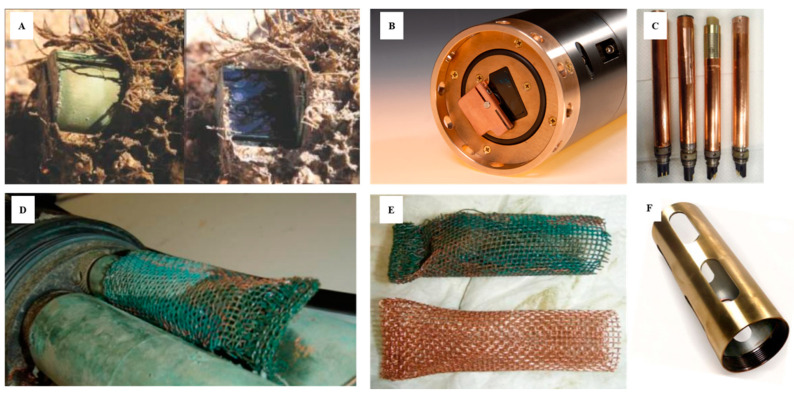
Sensor optics protected against biofouling by copper. (**A**) Copper shutter in the OBS501 Turbidity Probe [57]); (**B**) copper shutter + wiper in Water Quality Monitor (WQM) Wet-Labs and Sea-Bird Scientific sensor [50]; (**C**) sensor covered with copper tape; (**D**,**E**) antifouling copper “sock” used for conductivity sensors [81]; (**F**) copper guard [82] (reproduced with permission of Sea-Bird Scientific (Bellevue, WA, USA) and the permission of Campbell-Scientific (Loughborough, UK).

In recent years, some manufacturers have used this type of protection strategy. Some of them build the sensor head from this material, others incorporate copper protectors and guards as shown in Figure 5. With some manufacturers like YSI, a Xylem brand (Yellow Springs, OH, USA), these strategies are combined with the use of wipers. Some measuring systems even go further and develop a closed chamber as a “Copper Shutter” that generates a closed space through which to protect the optical windows (Figure 5). Sensors such as the Campbell Scientific OBS501(Loughborough, UK) have a specific shutter designed to protect the optics. The OBS501 is constructed to prevent sand grains or packed sediment from getting wedged between the shutter and sensor body, which inhibits the shutter’s movement. To do this, the OBS501’s shutter and body were designed to eliminate parallel surfaces between moving parts wherever possible. The probe also uses a flushing action that moves the sediment down and out of the cavity behind the shutter. This antifouling and cleaning system incorporated into the OBS501, called the ClearSensor Method [80], is able to sense whether the shutter motor is working harder than normal. If it is, the shutter moves slightly back and forth to dislodge the sediment before opening or closing completely. For additional protection, the company offers a plastic sleeve, as well as a copper sleeve that can provide additional protection, especially in sea water.

However, most sensors have exposed sensor heads. This eliminates many of the problems that can occur with pumping systems or shutter closures. In contrast to these open systems, protection against biofouling is more complicated. In some experiments on optical sensors carried out by Kerr and collaborator, a gel doped with biocides is used; however, there are problems with opacity affecting the performance once deployed [83].

### 4.3. Antifouling Coatings

Strategies to combat biofouling must be tailored to the type of sensor to be deployed. Coatings have become an attractive solution to reduction or prevention of biofouling. Coatings must be inert, facilitate diffusion and be transparent when applied to sensors requiring electrochemical or optical transduction.

#### 4.3.1. Non-Stick Coatings

Considerable attention in recent decades was focused on the creation of non-biocide, nontoxic coating systems that prevent the adhesion and settlement of fouling organisms. The aim of these foul release coatings is to create a surface that reduces the adhesion strength of the organisms. This causes organisms to detach by their own mass through the movement of the water [84]. Initial interest in the development of this type of compound has focused on materials such as fluoropolymers and siloxane elastomers and their copolymers because they combine low elastic modules with low surface energy. For example, hybrid xerogel compounds have been studied for their antifouling properties. It has been proven that they are able to inhibit the settlement of zoospores of the *Ulva* seaweed species [85].

Many of these compounds, such as silicones, could protect the sensor’s optical windows from scaling, even their surfaces, as they “release” macrofouling organisms when the hydrodynamic conditions are sufficiently robust [86]. This type of coating accumulates diatom silt, which is not released even at speeds above 30 knots in ships, so its viability in sensors for monitoring water quality would be in question because these are generally static elements. Three properties are particularly important in determining the success of such sensor fouling prevention coatings, notably: the surface energy of the coating, which influences the initial fouling; the modulus of the coating will determine whether the foulant is removed from the surface or some shear force; and the thickness of the coating, which determines the ease of removal of the foulant from the surface. However, this can have a negative effect on a sensor’s response. Materials such as polydimethylsiloxane (PDMS) have been shown to reduce contamination of fibre optic probes [87]. However, hyperbranched fluoropolymer (HBFP)-poly(ethylene glycol) (PEG) coatings have been identified for better antifouling performance [88]. Polymers containing phosphorylcholine have been shown to reduce the interaction of bacteria with medical devices due to the suppression of protein adsorption by phosphorylcholine at the surface, thus preventing the formation of surface-colonising biofilms that would correspond to the early stages of biofouling [89]. These coatings have the potential for sensor housings as these non-stick paints can be very useful if water currents are present to help minimise the establishment of biofouling. Some brands such as YSI, a Xylem brand (Yellow Springs, Ohio, USA), use nontoxic, non-water-soluble polymer nano-coatings in the form of a “C-Spray” to cover the exposed structures and surfaces.

#### 4.3.2. Biocide Coatings

Antifouling technologies using biocide coatings are based on the release of active compounds; these include contact leaching coatings, soluble/controlled depletion polymer (CDP) coatings and self-polishing copolymer (SPC) coatings. All these technologies have the common goal of controlled release of active molecules embedded in a polymer matrix [90].

- Contact Leaching Coatings use high molecular weight binders that are insoluble in seawater, such as vinyl, acrylic and chlorinated rubber polymers. They have a high mechanical resistance and can incorporate high quantities of toxic particles. The mechanism of release of these particles is gradual because the binder matrix is not soluble in seawater and the toxic agents contained in this matrix are released, leaving small pores through which seawater penetrates. Over time, the toxic particles remain in the deeper layers of the matrix in which they are immersed, making access to seawater difficult. This gradually decreases the rate of release of the toxic particles, causing the degree of protection to decline. The duration of the efficiency for this type of coatings is between 12 and 24 months providing potential for sensor use [91].
- Controlled Depletion Polymer is known as soluble ablative or eroding paints. These coatings, unlike the previous ones, contain biocides that are mixed with a nontoxic soluble matrix based on high amounts of rosin and its derivatives. In contact with seawater, the binder containing the biocide dissolves, gradually releasing the biocide. The limit of protection against biofouling is 12–15 months because the erosion rate of the matrix containing the biocide is too high from the moment the surface is laid out in the water [91]. However, the use of controlled depletion polymer (CDP) coatings can be reinforced with synthetic resins that are much stronger and more durable than rosin derivatives. The main difference with CDP coatings is that the process of release of the biocide occurs by hydration and dissolution not by hydrolysis [90].
- Self-Polishing Copolymer Coatings (SPC) are based on the use of acrylics or methacrylics that are easily hydrolysable in seawater. These copolymers mixed with biocides give smooth surfaces and are able to promote the leaching by controlling the erosion rate of the matrix [92]. These methacrylic copolymers function similarly to the methacrylic organotinic copolymers used in TBT-based paints but using copper, zinc or silicon ester groups instead. The mechanism of release of the biocidal particles has been extensively studied by Hellio and Yebra. Sea water diffuses into the insoluble matrix, causing the dissolution of the ester groups releasing the biocide particles due to their hydrolytic instability under alkaline conditions, such as those found in sea water [93].

Authors such as Wood and collaborators incorporated copper and cobalt phthalocyanines into a polystyrene resin [94]. These copper and cobalt metal complexes promote the formation of oxygen-free radicals from persulphates and peroxides. These reactive oxygen species cause damage at a cellular level. When the balance between these molecules and the antioxidant defence system that living beings possess is lost, oxidative stress is generated. In the case of the polystyrene resins mentioned above, the transition metals act as catalysts in the decomposition of the disinfectants of these compounds and the free oxygen radicals.

### 4.4. Electrochemical Antifouling Methods

An alternative to the use of fouling resistant coatings is to use electrochemistry. One of the methods that has been investigated by several groups is the generation of chlorine and hypochlorous acid by electrolysis of the water as a method for the prevention of fouling of marine sensors. This process occurs through an electrode adjacent to the sensor or through a conductive layer on the sensor surface. However, the disadvantage of this approach is that the coating can be physically degraded during the application of the potential in sea water [9].

**Figure 6:**
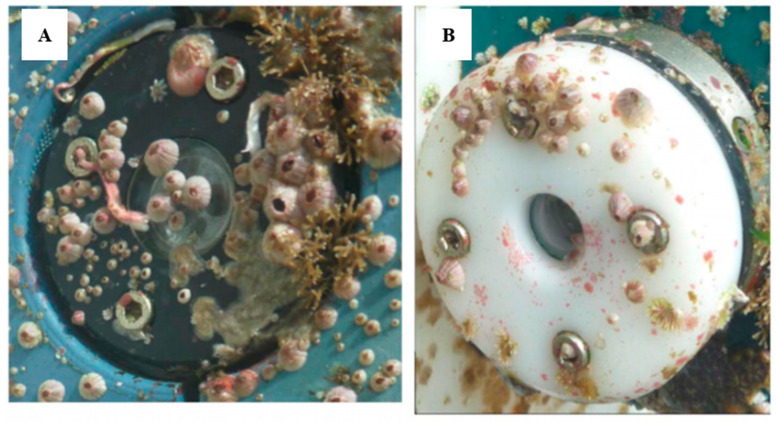
Antifouling system applied to optical sensor submerged in natural seawater for 6 months in France: (**A**) unprotected; (**B**) protected (© 2017 IEEE. Reprinted, with permission, from [98]).

Direct electrification of the organisms in different ways, by direct transfer of electrons from electrodes to the fouling organisms, has also been tested. Graphite-silicon electrodes [95] and titanium nitride (TiN) [96] have also been tested to combat biofouling. The application of electric pulses has been tested [97] in cooling systems. Electric pulses with amplitudes of the order of kV/cm, with durations of microseconds, proved to be effective on hydrazoans. Delauney et al. have tested biofouling protection in modified TriOS fluorometers [98] (Ammerland, Germany). They used a transparent conductive tin dioxide (SnO_2_) coating with optimised physicochemical properties by the CNRS-LISE UPR15 laboratory in collaboration with the Ifremer Technological Research Group in Brest, France [99,100] (Figure 6). This conductive layer acts as an anode, polarised at a specific potential, to produce chlorine. The SiO_2_ coating is produced by a pyrolysis process at 545 °C from a primary SnCl_4_ aerosol in combination with NH_4_F to form an F-doped film and subsequently from a second SnCl_4_ + SbCl_3_ aerosol to an Sb-doped film. A copper electrodeposition can then be made at the edge of the optical window to allow electrical contact with the SnO_2_ coating. These TriOS (Ammerland, Germany) fluorometers were modified by replacing their original optical windows with this fully integrated electrochemical arrangement and connected to the 12 V supply system of the sensor. Their total consumption in electrical terms was 1 mA.

Spears and Stone employed different methods based on copper screens [101]. These techniques based on galvanic anodes or sacrificial anodes are also employed as a corrosion protection system for buried or submerged metal structures. They are made of a metal alloy with a higher tendency to oxidize than the metal of the structure to be protected, with a more negative reduction potential. The potential difference between the two metals implies that the galvanic anode corrodes, preserving the structure to be preserved, since the anode material will be consumed in preference to the metal of the structure. This would inhibit growth in the structures while the surrounding areas would have significant marine fouling. Although this method is particularly effective in preventing barnacles and oysters from adhering to surfaces and is commonly used on ship hulls to protect propellers, the disadvantage is that it is expensive to install on sensors.

### 4.5. Irradiation to Combat Biofouling

#### 4.5.1. UV Radiation

**Figure 7:**
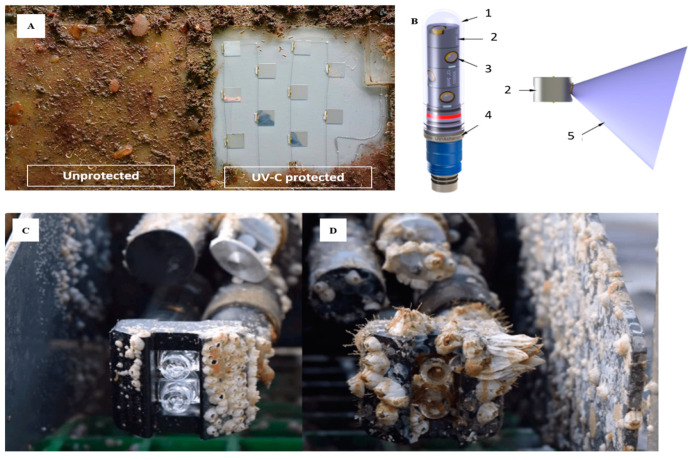
(**A**) Effectiveness of a UV-C irradiated panel versus the control panel against biofouling (RunWell-project, Royal Philips [108]). (**B**) UV Xchange major components [111]: (**1**) glass pressure case; (**2**) LED modules; (**3**) UV-LED; (**4**) titanium stem; (**5**) UV beam approximation in water (~70°). (**C**) UV-protected probe after nine months of deployment. (**D**) Non-UV-protected probe after nine months of deployment [109] (reproduced with permission of AML Oceanographic (and the permission of Royal Philips).

Ultraviolet irradiation techniques are being explored based on the effects that the wavelength of the ultraviolet spectrum (100–400 nm) has on the DNA of organisms. These techniques are known from the medical field for the disinfection and sterilization of instruments and work surfaces. They are effective since at the cellular level ultraviolet light is absorbed by the nucleic acids that lead to the formation of pyrimidine dimers and other lethal products [102]. The irradiation of surfaces by ultraviolet light has been investigated as a possible method to prevent biofouling in filtration membranes, marine sensors, industrial cooling systems, hospitals (to sterilize surfaces) [103], the disinfection of waste water in treatment plants [104,105,106] and the food processing industry, for control of foodborne pathogens and spoilage organisms for food safety and shelf-life extension [107]. The irradiation of surfaces by ultraviolet light has been investigated as a possible means of preventing biofouling in filtration membranes, marine sensors, etc. The advantage of such a method is that it prevents the lixiviation of toxic compounds into the sensor’s larger environment. However, until recently its incorporation into sensors had not been practical due to the high energy requirements. Companies such as Royal Philips (Amsterdam, Netherlands) are working to develop a new technology that uses Ultraviolet-C (UV-C) emission panels applied to submerged surfaces to keep the area clean [108] (Figure 7A). This new approach, although still experimental, appears to have promising results on boat hulls; its application in sensors is not yet well developed and only a few manufacturers such a AML Oceanographic (Dartmouth, Canada) or Mariscope Meerestechnik (Kiel, Germany), in collaboration with the Leibniz Institute for Baltic Sea Research (IOW), are using this technology to keep the surface of sensors clean [109,110]. The UV-Xchange plug-in system from AML Oceanographic (Dartmouth, Canada) (Figure 7B) allows the sensors to perform their full potential over the duration of long-term on-site deployments [111]. Installed directly into the end cap of an X-Series instrument, the module can be adjusted to various positions, allowing optimal coverage of all sensors requiring protection. Other manufacturers, such as Chelsea Technologies (Molesey, UK), also use UV-C irradiation technology, but unlike AML Oceanographic this system is incorporated within the sensor housing. This UV technology can be found in their VLux-series fluorometers [55].

#### 4.5.2. Photocatalytic Materials

Another option is to coat the surface with a photocatalytic material, such as TiO_2_ and WO_3_, which inhibits algae growth [112]. TiO_2_ has been widely used for hydrolysis-induced self-cleaning surfaces due to its favourable physical and chemical properties [113]. This compound can also show photocatalytic and superhydrophilic photoinduced properties. Under UV irradiation, TiO_2_ surfaces become progressively superhydrophilic, which makes them effective in combating marine fouling [114]. Other studies also confirmed the same phenomenon for ZnO [115,116,117,118]. This UV-induced variation in the surface structure of oxides has been used in the manufacturing of self-cleaning windows by Pilkington (Tokyo, Japan) (“Activ glass”) and PPG Industries (Pittsburgh, PA, USA) (“SunClean glass”). In the presence of sunlight, nanometric TiO_2_ particles act as photocatalysts and at the same time increase the wettability. The photocatalytic process helps to decompose the organic material deposited on the surface and the increased wettability improves the removal of the loose particles by the movement of the water itself [114]. Although the application of these types of coatings on sensors is non-existent, it could be an alternative to be explored for the coating of optical lenses or windows and protecting them from marine fouling.

#### 4.5.3. Laser Irradiation

Another solution in the experimental phase is laser irradiation as a means of preventing biofouling by barnacles and diatoms. The ability of pulsed laser irradiation to cause damage to biofouling organisms has been recently investigated. Nandakumar and collaborators carried out pulsed laser irradiation studies on two different species of diatoms, *Skeletonema costatum* and *Chaetoceros gracilis* [119]. The results showed that when exposed to low-power laser irradiation, these showed mortalities of between 53% and 98%, respectively, for exposure times of 2 and 300 s. It can be seen that the mortality increased with increasing duration of the laser irradiation. The estimation of the chlorophyll concentration in the irradiated samples showed a considerable reduction, varying between 9.8% and 57% in *C. gracilis* and 3% and 70.3% in *S. costatum*, for 2 and 300 s of irradiation, respectively. The study showed that low-power pulsed laser radiation can cause significant damage to at least these two species of planktonic diatoms, which may be a step forward in combating the first phases of the biofilm formation process that will lead to biofouling in which diatoms, among others, participate. Whelan et al. also showed an increased efficiency of the laser kill rate by increasing the density and duration of the laser energy [120]. These active techniques present potential as they do not generate any type of biocide; however, the main energy requirement to operate these in autonomous systems is too high to be an effective solution for autonomous sensors [11].

#### 4.5.4. Ultrasonic Irradiation

The efficiency of ultrasonic irradiation to control biofouling has been investigated by several groups as an effective method to reduce biofouling. Studies on the control of biofouling using ultrasonic techniques have been reported in the literature [121,122,123,124,125]. In this approach, the cavitation phenomenon developed by high-intensity ultrasound is likely to be responsible for the control of biofouling. Cavitation creates high liquid shear forces that prevent the settlement of organisms on the submerged surfaces, followed by a violent implosion [123]. It has been shown that the vapour bubbles generated during cavitation can damage the cells and micro-organisms that produce biofouling. These methods have been used by the United States Navy in oceanographic sensors [126]. The use of low frequency sound to prevent zebra mussel fouling on structures was studied by Donsky and Ludyanskiy [127]. It was found that the combined effect of sound and vibration destroys zebra mussel larvae and waterborne sound prevents juvenile and adult mussels from settling and translocating onto exposed surfaces Although these techniques are effective in combating biofouling on large surfaces, such as boat hulls, the power limitations of a stand-alone sensor that is usually battery-operated make it an unfeasible method at present.

#### 4.5.5. Biomimetic Antifouling Strategies

One of the most promising approaches to developing materials to combat biofouling is to borrow ideas and concepts provided by nature and turn them into technologically elegant and, above all, environmentally friendly solutions. The approach to designing textures and materials based on those found in nature is known as biomimetics. This concept is based on the use of material characteristics present in nature to inspire novel improvements in existing engineered materials, or even to imitate ideas from nature to produce completely new solutions to engineering problems [128,129]. The design of novel antifouling solutions using biomimetics is an attractive prospect, as many marine organisms appear to have some intrinsic ability to resist epibiosis by both chemical and physical means [130].

These strategies can be very smart and promising in reducing marine fouling by the control of the colonisation of fouling organisms. According to Chambers et al. (2006), there are more than 160 antifouling products derived from organism such as algae, sponges and bacteria, among others [21]. These biocomposites can be used in marine sensors as part of the composition of the paints or coatings used for these purposes, allowing the elimination of synthetic biocides that are often much more harmful to the environment; for example, the compounds of the bacteria of the genus *Pseudoalteromonas*, despite having an effectiveness of 14 days due to its fragility. The use of genetic engineering could transfer the gene to another genus of bacteria that is much more resistant and produce coatings with a long life in terms of antifouling properties [9].

#### 4.5.6. Fouling Management on Sensors

Biofouling protection must be done carefully on autonomous sensors so that it does not interfere with the measurements. In the case of fluorometers, the use of biocides generated by electro-chlorination can affect the measurements [131]. Active systems, such as wipers for optical windows, should not be activated when the measurement is being taken. These solutions must remain off when the measurement is being made, allowing energy saving. There must be a balance between the “on cleaning mode” and the “energy saving mode”, depending on the biofouling pressure.

#### 4.5.7. Summary of the Current State of the Art

To date, sensor manufacturers have employed a wide range of strategies to mitigate fouling. A summary of the strategies used is presented in Table 2. The criteria for the selection of implemented strategies is driven by sensor application, environment, user requirements in terms of analytical performance and the underlying sensor’s operating principles. Sensor application has been the main criteria to date. Sensors operating in coastal and oceanic zones come equipped with the most innovative and advanced antifouling strategies (see Table 2, YSI, a Xylem brand, Yellow Springs, Ohio, USA and Sea-Bird Scientific, Bellevue, Washington, USA). Other sensors for environmental applications like fresh water and source water monitoring use fewer complex approaches and rely mostly on wipers and coatings. The latter category of sensors applied to the monitoring of drinking water, waste water and industrial process water rely mostly on stand-alone strategies in the form on cleaning in place attachments based on pressure jet or ultrasonic cleaning, such as the TriOs FlowCell FC 48/10 Ultrasonic system [132] (Ammerland, Germany). For this type of application, antifouling requirements are not limited by power, accessibility to site or maintenance. When such sensors are implemented in environmental monitoring, such as agricultural catchments, the same antifouling strategies are used. In this case the sample is pumped to a flow-through tank equipped with sensors; however, the overall infrastructure required can be both extensive and expensive. Another criterion that is driving innovation is based on the sensor’s operating principle. For marine and coastal observations, sensors can be delimited into three categories: acoustic, optical and electrode based [12]. Acoustic sensors have the highest tolerance to biofouling and are only affected by excessive macrofouling in the form of structural damage and range, hence they are not covered in this review [12]. Optical sensor currently in use can be split into two categories, solid state and hybrid membrane-based sensors. For the former, the active sensing area at the interface with the environment is usually an optical guide in the form of optical windows, lenses or optical fibre ends. Sensors relying on this approach use particle light scatter, absorption and fluorescence to measure parameters like turbidity, Chl-a, rhodamine, nitrate, etc. The latter category relies on gas diffusion membranes at the interface and an underlying analyte recognition layer. An example of such sensors is the optical dissolved oxygen probe. Electrode-based sensors, similar to optical sensors, can incorporate membranes (i.e., ion-selective electrodes like pH, dissolved oxygen, etc.) or not (temperature and conductivity). Among them, the electrode and membrane-based sensors are the most susceptible to data drift and inoperability due to biofouling. Due to the fact that membranes cannot be coated with either biocide or non-stick coatings, the fouling protection on such active surfaces has relied on strategies like wipers, light blocking, biocide injection and passive inhibitors (Table 2). Optical sensors, on the other hand, have been more resilient to data drift and fouling even though these sensors have zero tolerance to fouling. The main reason for this has been the ease of keeping such surfaces clean. Made of fused silica, sapphire or quartz, optically active surfaces have high robustness, tensile strength and high chemical compatibility. These properties allow the use of wipers, high pressure jets and ultrasonication without affecting the integrity of the surface. In addition, the surfaces are smooth with very low roughness, which discourages adhesion and sticking, and transparent coatings can be used to provide additional protection (see TriOS, Ammerland, Germany, Table 2). From a physical design perspective, the majority of sensors contain a sensor housing and an active measuring zone (optical head, sensing probes, etc.). The measuring zones can differ greatly based on the type of sensor (single vs. multi-parameter), measuring principle and sampling regimes. Implemented strategies to date have focused almost exclusively on the active sensing areas (i.e., optical windows, membranes and conductivity cells) and on the components in the immediate vicinity to them (wipers, probe housing, mounting plates, guards, etc.). The most successful solutions implemented use strategies in tandem for both the active surfaces and the components in the immediate vicinity. Examples include the combination of wipers with biocidal materials (mainly copper, copper alloys and copper-based paints) or the combination of wiper/shutter systems with bleach injection, TBT controlled release and biocidal materials (copper components) (Table 2). Such approaches limit excessive growth and biofouling build-up, ensuring the optimal performance of the wipers, shutters and in situ injection systems, and ultimately the effective protection of the active surfaces.

The variety of sensor types, their deployment scenarios and application preclude a single antifouling strategy. To date, there is no available universal strategy that is effective, but rather a combination of strategies that has extended deployment times and free maintenance periods from days to months. From a business perspective, state-of-the-art, relatively expensive multi-parameter sensors come better equipped to tackle fouling. The performance of these sensors absorbs the research and direct costs associated with the implementation of integrated antifouling strategies. On the other hand, lower-cost sensors, generally single-parameter sensors, have less antifouling protection. Some manufacturers provide options for the use of stand-alone wipers, tape or paint at an added cost, which often times can be prohibitive, while others leave the choice to the end-user. From a sensor design point of view, manufacturers operating in coastal and marine environments place the antifouling strategy at the center and build the sensor around it (see the WQM sensor from Sea-bird (Bellevue, Washington, USA) and Wet-Labs (Philomath, Oregon, USA Table 2, or the wiped temperature/conductivity probe, from YSI, a Xylem brand, Yellow Springs, OH, USA [38]).

#### 4.5.8. Current Limitations and Future Directions

Water monitoring instrumentation plays an increasingly important role in current societal issues such as energy management, ecosystem health, raw materials of the ocean and the ocean’s impact on climate, weather and food security [10]. Sensors in industrial, municipal and environmental monitoring are advancing our understanding of science, aiding developments in process automatization and control and support real-time decisions in emergency situations. For sensors to become commonplace, the data they provide must be robust and reliable at an acceptable financial burden. One of the main limitations of water monitoring sensors to date is fouling, either in the form of physical or biological fouling, which in turn affects the integrity of the data and increases operational costs. Current state-of-the-art antifouling strategies for environmental monitoring have been able to extend deployment times and reduce operational cost. However, the progress is still less than ideal and antifouling strategies have to catch up with the rapid advancements in sensors. Such advancements have been mainly catalysed by recent progress in photonics, material science, data communication protocols and data analytics, power management and storage, energy harvesting and manufacturability. Sensors are become smaller, smarter, increasingly specialised, available for more parameters and cheaper. The vision of coastal sensor networks for real-time decision support and marine networks for ocean observations is increasingly tangible. Advanced deployment platforms now exist to support various monitoring needs and include buoys, mini buoys, autonomous surface vehicles, buoyance engine vehicles (floats and gliders) and thruster-driven subsurface vehicles [10,133]. In this context, sensor antifouling strategies play a critical role in present and future sensor networks.

To date, two main trends are noticed among sensor manufacturers. Manufacturers of high-specification instrumentation are placing the antifouling strategy at the centre of product design as an essential performance indicator. This translates into smartly engineered integrated solutions that extends the deployment periods and target end-user requirements. Such sensors are the result of many years of experience, in-house know-how and research and continuous engagement with end-users. These types of manufacturers also provide specialised, tested stand-alone antifouling strategies in the form of accessories (wipers, antifouling sprays, tape, etc.). It is likely these manufacturers will continue to rely on integrated proprietary antifouling strategies and engage actively in research and development, while implementing the solutions of the future. The second trend noticed, is the emergence of lower-cost, single-parameter or niche-market sensors. For manufacturers of these sensors, the antifouling strategy is not a critical design constrain. There are manufacturers that include partial passive solutions like copper-based components, copper-based paints and coatings and non-stick coatings for optical windows. Some go further, and use integrated emerging strategies like UV light sources, either purposely fitted or already present (inherent) and used as the light source for the optical detection (see Chelsea Technologies, Molesey, UK, Table 2). Other manufacturers rely on the end-user to implement the antifouling strategy or collaborate with specialised companies for the implementation of active protection. Such companies are now emerging, and offer wiper-based solutions that can be fitted to a wide range of optical instrumentation [56] or UV radiation solutions [109,110,111]. Going forward, it is likely that manufacturers that aim to keep the cost low will avoid the integration of moving parts in their sensors (i.e., wipers, shutters and pumps) and rely on low-cost solutions and external sources.

Among the strategies under development, UV radiation-based approaches show the most promise for future implementation. This technology showed great potential in experimental studies, but until recently its application has been limited by the availability of stable, affordable, low-power light sources. With UV LEDs becoming increasingly available for the lower UV region, cheaper, more reliable and powerful the application of this strategy is likely to be. UV-protection solutions are already available on the market from specialised companies [111]. To date, these solutions target the active surface areas of the sensors, particularly the optical windows, with the radiation being focused on the components of interest from the outside. It is likely future developments will see the integration of LEDs into sensors at the design stage, with LEDs being placed internally behind UV transparent optical windows, or strategically positioned to cover the areas of interest. The application of UV LEDs can extend not only to the critical active surfaces but also the components adjacent to them and even sensor housings or deployment platforms. To that extent, research is already on-going looking at the potential for integration in ship hulls [108,134,135]. UV radiation has the potential to replace the two main strategies used to date for active surface areas, namely the wiper-based and the wiper/shutter/biocide injection-based systems. These systems provide both physical and biological protection, while the performance of UV radiation in terms of physical fouling protection is not clear. Future strategies might incorporate UV radiation coupled with non-stick foul release or self-polishing coatings to match the performance of exiting systems, at a much lower cost. Although Teflon-based coatings are already in use for temperature-based sensors, future research should focus on the development of transparent, nontoxic approaches that can be applied to optical surfaces without affecting the analytical performance of the instrument. Such research is already on-going, and the future might see coatings available on the market. The use of biocide-based materials and coatings is likely to continue in the future and even expand. Research and development on copper-based solutions, in the form of cheaper and higher performance copper alloys, paints and coatings, could further expand the application of these strategies. On the other side, coatings and materials incorporating new and innovative biocide materials must pass strict regulatory requirements before they are made available on the market. Development and testing take many years and is costly, which is likely to hamper research interest. The design of novel antifouling solutions using biomimetics is an attractive prospect. Although at an incipient stage, nature-inspired engineered surfaces on sensor components, housings and deployment platforms could become a reality in the future. Another solution that shows potential is the implementation of self-calibration protocols to account for data drift due to fouling [136]. With the rapid developments taking place in photonic integrated circuits and in situ data processing capabilities, data analytics is becoming an essential component of sensor networks. To that end, data analytics can be used to extract the features in the sensor data associated with fouling build-up and smart protocols can be developed to allow automated/unattended self-calibration of the sensors. Such capabilities could in theory extend deployment times, yet still provide robust, reliable data.

## 5. Conclusions

Antifouling strategies play a critical role in present and future aquatic sensor and sensor networks. For submersed instrumentation, biofouling is the single biggest factor affecting the operation, maintenance and data quality. This increases the cost of ownership in that it is prohibitive to maintain operational sensor networks and infrastructures. Despite the importance of protection against biofouling for in situ instrumentation, progress towards an ideal operational strategy has been slow. Although many techniques have been developed and tested in the last decade, very few of them have been implemented commercially. To date, there is no available universal strategy that is effective, but rather a combination of strategies that has extended deployment times and free maintenance periods from days to months. The most notable developments and advancements have come from industry, with the development of specialized, tailor-designed solutions in the form of mechanical wiper, biocide injection, pressure jet and ultrasonication systems.

In the race for coastal sensor networks for real-time decision support and marine networks for ocean observations, sensors are become smaller, smarter, increasingly specialized and diversified and cheaper. Advanced deployment platforms now exist to support various monitoring needs together with state-of-the-art power and communication capabilities. The antifouling strategies of the future have to be low cost, robust and highly efficient to allow for long-term continuous monitoring at large spatial scales.

For immediate implementation, UV radiation shows the most promise for becoming a reality. The solution is attractive for its simplicity, low power requirements, potential for integration and moderate to low cost. With UV LEDs becoming increasingly available for the lower UV region, a cheaper, more reliable and powerful application of this strategy is likely to expand. Combined with anti-stick coatings and copper-based materials, UV-based strategies could provide an ideal solution, matching or even outperforming the current mechanical-based systems. Further research is needed to answer these questions and industry plays a critical role in driving it, either through independent research or collaborative programmes. Non-stick, self-polishing and advanced biomimetic coatings and materials, together with self-calibration protocols, are promising strategies for implementation in the near future. Although some of these strategies are spuriously used, further fundamental research is required to advance the knowledge the underlying biofouling mechanisms, which, in turn, could underpin the next generation of antifouling solutions for sensor technologies.

## Figures and Tables

**Table 1:** Materials used in the sensor body, connections and sensor head.

Material	Sample Use of Material in a Sensor	Sensor Type or Application
**Metals**		
Titanium	Sensor housings	Available in commercial turbidity sensors
Anodised aluminium	Sensor housings	All, freshwater applications
304L Stainless steel	Sensor housings	Specifically, marine applications and corrosive industrial applications
316L Stainless steel	Filtration	Available for particulate matter screening on some conductivity ad temperature sensors
Stainless steel microscreens	Sensor housings	Replacement for SS housings
Copper	Antifouling	Most commercial systems
**Plastics**		
Polyoxymethylene (Acetal, Delrin^®^ (Wilmington, DE, USA)) [26]	Sensor housings	Available on commercial pH, fluorimetry and ORP sensors
Polyphenylene sulphide (PPS) (Ryton^®^) (Bollate, ITALY) [27]	Sensor housings	Some pH and ORP sensors
FEP Teflon	Membranes	Dissolved oxygen
Polyurethane	Cables	Most
Acrylonitrile butadiene styrene (ABS)	Sensor housings	Some models; OTT Orpheus Mini
HD polyurethane	Cables	Most
Poly Vinyl Chloride (PVC)	Cables	Most
Cross-Linked Polyethylene (XLPE)	Cables	Most
Chloroprene Rubber (CR)	Cables	Most
Polyurethane (PUR)	Cables	Most
**Other Materials**		
Epoxy resins	Electronics, housing material	Most
Silicon	Diaphragms	Water level sensors
Sapphire	Optical windows	Turbidity
PVDF membranes	Filtration membranes	Phosphate, combined models
Glass	Optical windows	Turbidity
Fused Silica	Optical windows	Most

**Table 2:** Antifouling strategies implemented by sensor manufacturers.

**Manufacturer**	Sensor Type	Model	Antifouling Strategy	Protected Component	Reference
TriOS	Photometer	TriOS VIPER	Coatings, ultrasonication cell	Optical window	[39]
	Fluorometer	TriOS enviroFlu	Coatings	Optical window	[40]
		TriOS nanoFlu	Coatings	Optical window	[41]
Sea-Bird Scientific	Fluorometer	Sea-Bird Scientific ECO Fluorometer	Wiper + copper plate	Optical head and optical windows	[42]
	Scattering	Sea-Bird Scientific ECO Scattering Sensor	Wiper + copper plate	Optical head and optical windows	[43]
	Combined scattering and fluorescence	Sea-Bird Scientific ECO Triplet	Wiper + copper plate	Optical head and optical windows	[44]
	Multi-parameter: CTD, ODO, pH	Sea-Bird HydroCAT-EP	Active flow control, passive flow prevention, light-blocking, active biocide (TBTO) injection, passive inhibitors and copper faceplate and wiper	Optical head. optical windows, sensor housing, conductivity cell and temperature probe	[45]
	Hyperspectral Radiometer	Sea-Bird Scientific HyperOCR Radiometer	Copper shutter	Optical window	[46]
	Multispectral Radiometer	Sea-Bird Scientific ECO PAR	Wiper + shutter Copper plate	Optical head and optical windows	[47]
		Sea-Bird Scientific OCR	Copper shutter	Optical windows	[48]
Sea-Bird and Wet-Labs	Combined fluorometer-turbidity and CTD	Wet-Labs and Sea-Bird Scientific WQM	Active flow control, passive flow prevention, light-blocking, active biocide (TBTO) injection and passive inhibitors	Optical head. optical windows and sensor housing	[49,50]
YSI, a Xylem brand	Multiparameter-modules	YSI EXO-series	Central Wiper, copper guard, copper sleeves/mesh, antifouling sleeves for overall sensor body, antifouling spray	Optical head, optical windows and sensor housing	[51]
		YSI 6 series	Probe wiper, copper sleeves, copper alloys	Optical head and optical windows	[52]
Photonic Measurements	Spectrometer	UV254 Probe	Pressurised water cleaning	Optical window	[53]
EFS	Multiparameter UV-probe	COD UV-Probe 254+	Compressed air-module	Optical window	[54]
Chelsea Technologies	Fluorometer	VLux Algae Pro	UV light, copper bezels and Hydro-Wiper	Optical window	[55,56]
Campbell Scientific	Turbidity meter	OBS501	Shutter/wiper mechanism + biocide chamber + copper alloys	Optical window	[57]
Turner Designs	Fluorometer	C3	Copper tape + mechanical copper wiper	Optical window	[35]
	Fluorometer	C6P	Copper tape + mechanical copper wiper	Optical window	[36]
Hydrolabs	Multiparameter-modules	DS5X	Central Wiper, copper guard, copper mesh, copper tape	Optical head, optical windows, pH and temperature probes	[58]
S::can	Spectrometer	Spectro::lyser V3	Compressed air or brush	Optical window	[59]
